# A pair-wise meta-analysis highlights circular RNAs as potential biomarkers for colorectal cancer

**DOI:** 10.1186/s12885-019-6136-9

**Published:** 2019-10-15

**Authors:** Chen Li, Xinli He, Lele Zhang, Lanying Li, Wenzhao Zhao

**Affiliations:** 1 Department of Traditional Chinese Medicine, The First Affiliated Hospital, and College of Clinical Medicine of Henan University of Science and Technology, Luoyang, 471000 Henan Province China; 2 Department of Gastrointestinal Surgery, The First Affiliated Hospital, and College of Clinical Medicine of Henan University of Science and Technology, No.24 Jinghua Road, Jianxi District, Luoyang, 471000 Henan Province China

**Keywords:** Circular RNA, Colorectal cancer, Diagnosis, Prognosis, Clinicopathologic association, Meta-analysis

## Abstract

**Background:**

Circular RNAs (circRNAs) have emerged as a special subset of endogenous RNAs that are implicated in tumorigenesis and cancer progression. Herein we aim to carry out a meta-analysis to evaluate the clinicopathologic, diagnostic and prognostic significance of circRNA expression in colorectal cancer (CRC).

**Methods:**

A systematic search of online databases was performed for original articles published in English, which investigated the diagnostic accuracy, prognostic utility, and clinicopathologic association of circRNA(s) in CRC. Data were strictly extracted and study bias was judged using the Quality Assessment for Studies of Diagnostic Accuracy II (QUADAS II) and Newcastle-Ottawa Scale (NOS) checklists.

**Results:**

A total of 13 studies, involving 1430 patients with CRC, were included in the meta-analysis. The clinicopathologic study showed that abnormally expressed circRNAs were correlated with tumor diameter (*P* = 0.0350), differentiation (*P* = 0.0038), lymphatic metastasis (*P* = 0.0119), distant metastasis (*P* < 0.0001), TNM stage (*P* = 0.0002), and depth of invasion (*P* = 0.001) in patients with CRC. The summary area under the curve (AUC) of circRNA for the discriminative efficacy between patients with and without CRC was estimated to be 0.79, corresponding to a weighted sensitivity of 0.77 [95% confidence interval (CI): 0.74–0.79], specificity of 0.67 (95%CI: 0.64–0.70), and diagnostic odds ratio (DOR) of 7.52 (95%CI: 4.66–12.12). Survival analysis showed that highly expressed circRNAs were correlated with significantly worse overall survival (OS) [hazard ratio (HR) = 2.66, 95%CI: 2.03–3.50, *P* = 0.000; *X*^2^ = 4.34, *P =* 0.740, *I*^2^ = 0.0%], whereas lower expression of circRNAs was associated with prolonged OS (weighted HR = 0.30, 95%CI: 0.17–0.53, *P* = 0.000; *X*^2^ = 1.34, *P* = 0.909, *I*^2^ = 0.0%). Stratified analysis in circRNA expression status, and test matrix also showed robust results.

**Conclusion:**

Abnormally expressed circRNAs may be auxiliary biomarkers facilitating CRC diagnosis, and promising prognostic biomarkers in predicting the survival of CRC patients.

## Background

Colorectal cancer (CRC) is a leading cause of cancer-related morbidity and mortality worldwide [1]. In China, the incidence and mortality rate of CRC are ranked fourth and third of all malignant tumors, respectively, and the incidence rate of CRC is increasing year by year [2]. Patients with CRC have an unfavorable prognosis; however, the prognosis of CRC is better when the disease is diagnosed in the early stages [3]. Routine blood biomarkers are not powerful enough to aid diagnosis or predict prognosis in patients with CRC [4]. Therefore, the development of novel diagnostic and prognostic biomarkers is crucial to reduce CRC-related deaths.

Recent advances in novel genetic and epigenetic biomarkers for the management of CRC have provided new research perspectives. Circular RNAs (circRNAs) are non-coding RNA molecules that lack a 5′-terminal cap and 3′-terminal poly A tail [5]. CircRNAs are abundant in cells and tissues and their unique sequences endow them with special biological properties such as cytoplasmic microRNA sponges, attaching elements of RNA-binding proteins, or nuclear transcriptional regulators [6, 7]. The formation of a covalently closed continuous loop also makes circRNAs more stable than linear mRNAs [8]. In recent years, circRNAs have been highlighted as novel biomarkers for the management of CRC [9–21], but the findings remain controversial. The present study summarizes the clinicopathologic, diagnostic, and prognostic significance of circRNAs in CRC patients via a meta-analysis.

## Methods

### Literature search

This study was conducted in accordance with the Preferred Reporting Items for Systematic Reviews and Meta-Analyses (PRISMA) Checklist issued in 2009 [22]. Online databases including PubMed, EMBASE, Web of Science, SCOPUS, and Chinese National Knowledge Infrastructure (CNKI) were searched for eligible studies that evaluated the diagnostic, prognostic or clinicopathologic significance of circRNA(s) in CRC. The following search terms were used with different combinations in different databases: “colorectal cancer”, “colorectal carcinoma”, “colorectal neoplasms”, “carcinoma of colon”, “circular RNA”, “circRNA”, “hsa circ”, “clinicopathologic feature”, “clinicopathological characteristics”, “clinicopathological parameters”, “diagnosis”, “diagnoses”, “sensitivity”, “specificity”, “area under the curve”, “AUC”, “ROC curve”, “prognosis”, “prognoses”, “hazard ratio”, “overall survival”, “OS”, and “HR”. Patients with CRC were considered the “case group”, whereas those with benign lesions or healthy individuals were considered the “control group or controls”.

### Study selection

Inclusion criteria were: (1) original research reporting the diagnostic accuracy, or prognostic utility, or clinicopathologic association of single or parallel circRNAs in CRC; (2) the diagnosis of CRC was histopathologically confirmed; and (3) studies investigating the clinical utility of circRNA(s) in CRC, with sufficient data to plot the 2X2 table, or with available HR values and 95% confidence interval (CI), or available *P* values for clinicopathologic associations. Exclusion criteria were: (1) articles not published in English; (2) reviews, basic studies, comments, meta-analyses, letters or case reports; and (3) studies defined as low quality.

### Data extraction

Two authors each assessed the eligibility of all studies and extracted the data. The following data were extracted from each study: (1) baseline information including name of first author, date of publication, number of cases, control source, test matrix, method, reference gene, cut-off point, circRNA(s) type and expression status; (2) clinicopathologic information (as *P* values) regarding circRNA(s) expression and age, gender, cancer location, tumor diameter, differentiation, serosal invasion, lymphatic metastasis, distant metastasis, and TNM stage; (3) diagnostic data including sensitivity, specificity, area under the curve (AUC) value, or the true positive (TP), false positive (FP), false negative (FN), and true negative (TN) values; and (4) prognostic data including duration of follow-up, HR value and 95%CI for OS.

### Quality assessment

Study quality in relation to diagnosis was rated in accordance with the Quality Assessment for Studies of Diagnostic Accuracy II (QUADAS II) checklist, which comprises seven questions regarding patient selection, index tests, reference standards, flow, and timing [23]. Risk of bias was rated as “no”, “yes”, or “unclear”, and only an answer of “yes” received a score of 1, otherwise no score was awarded. Study quality in relation to prognosis was judged by the Newcastle-Ottawa Scale (NOS) [24], wherein the risk regarding study selection, comparability, and outcome were assessed. A study was deemed to be of high quality when the QUADAS II score was ≥4 stars, and ≥ 6 stars for the NOS checklist [25].

### Statistical analysis

STATA software (version 12.0) was used to analyze the clinicopathologic and prognostic significance. Meta-Disc (version 1.4) was utilized to summarize the weighted diagnostic parameters including sensitivity, specificity, positive likelihood ratio (PLR), negative likelihood ratio (NLR), overall diagnostic odds ratio (DOR) and AUC. Heterogeneity among studies was assessed by the *X*^2^ and inconsistency *I*^2^ (I-square) tests, and the cut-off point was set as *P* < 0.05 in the *X*^2^ test or *I*^2^ > 50%. Associations between circRNA expression and clinicopathologic parameters were determined using the *P* values combined with Fisher’s test [26]. HR and 95%CI were combined based on multivariate Cox hazard regression analysis, and the random effect model was chosen when significant heterogeneity was observed. Sensitivity and meta-regression tests were used to identify the underlying causes of heterogeneity [25]. Publication bias was quantitatively judged by Deeks’ funnel plot asymmetry test, Begg’s and Egger’s tests, and *P* < 0.05 was considered statistically significant.

## Results

### Search results

**Fig. 1:**
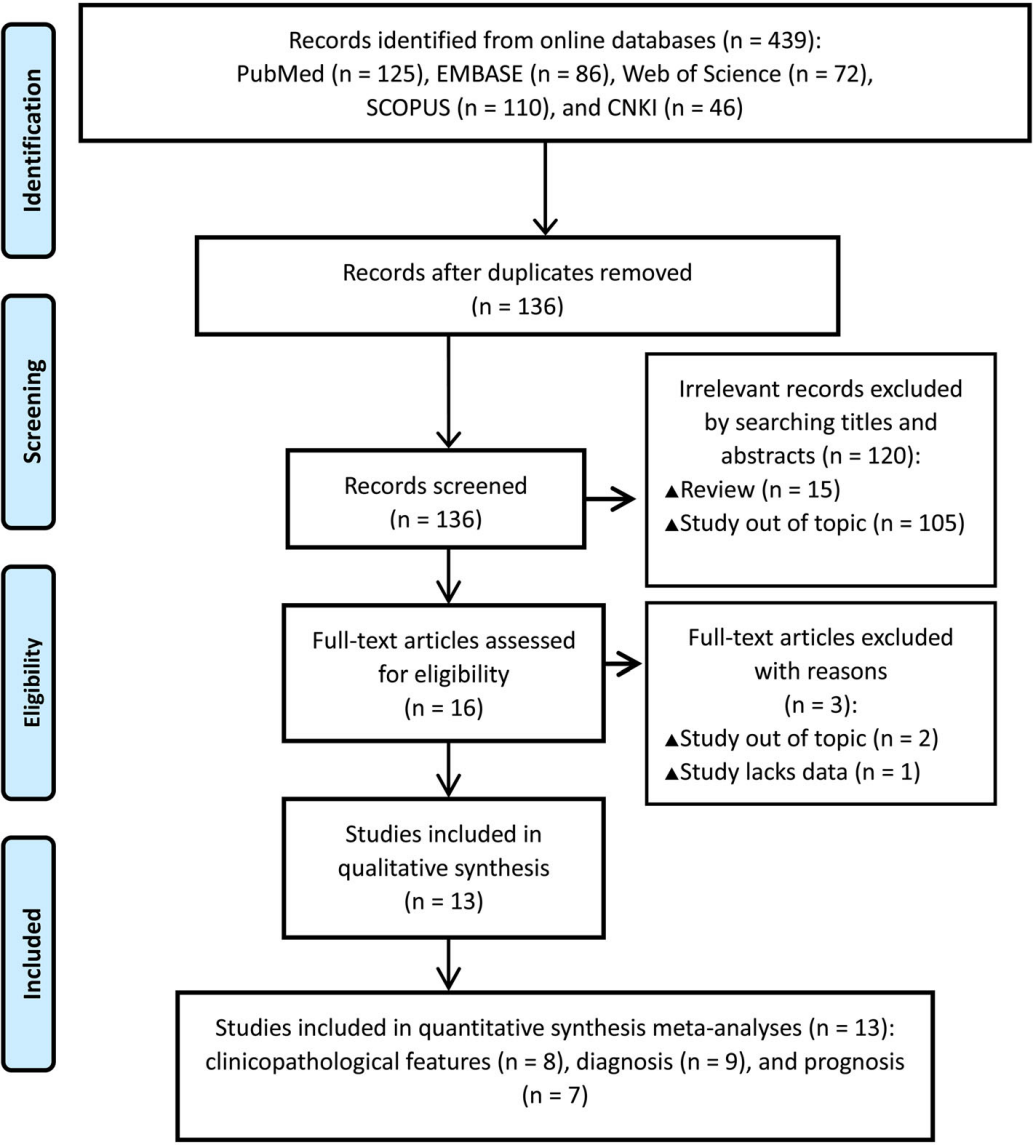
Flow chart of the study search strategies

The study selection procedure is shown in Fig. 1. In the initial search, a total of 439 publications retrieved from PubMed, EMBASE, Web of Science, SCOPUS, and Chinese National Knowledge Infrastructure (CNKI) databases seemed to meet the inclusion criteria. Of these, 303 publications were identified as duplicates and were eliminated. After reading article titles and abstracts, 120 records were eliminated as no association between circRNA expression and CRC was described or the articles were reviews. In the full-text verification, 16 articles were excluded as the studies were out of topic or lacked sufficient data. Finally, 13 studies were included in the quantitative meta-analysis.

### Study characteristics and study quality

The included 13 studies comprised eight studies on clinicopathologic parameters [9–11, 13, 14, 16, 19, 20], nine on diagnosis [9–14, 16, 19, 20], and seven on prognosis [10, 12, 14, 15, 17, 18, 21]. The baseline characteristics of all included studies are summarized in Tables 1 and 2. All 13 studies were carried out in Asia. A total of 1430 CRC cases were included, and the sample size ranged from 32 to 318. All CRC cases were diagnosed by histological and pathological examinations. The tissue samples were obtained prior to clinical treatment. circRNA expression level was determined using quantitative real-time polymerase chain reaction (qRT-PCR) or RNA sequencing, and the reference genes included *GAPDH* [10–18, 20, 21], *18S rRNA* [9], and *U6* [19]. Six types of circRNAs were recognized as tumor promoters [12, 15, 16, 18, 19, 21], and seven were tumor suppressors [9–11, 13, 14, 17, 20]. For survival analysis, the follow-up period was available in two studies, and three articles contained data on HR and 95% CI, whereas HR values in the remaining four articles were unclear and were calculated indirectly.

**Table 1:** Main characteristics of the meta-analysis for diagnostic performance and clinicopathologic association of circRNAs in CRC

Study	Locale	Patient number	Control number	Control type	Sample Type	CircRNA signature	Expression status/Biological function	Method	Cut-Off Value	Reference gene	AUC	Assessed clinicopathologic association
Wang J 2018 [9]	Chinese	102	102	Adjacent noncancerous tissues	Tissue	*hsa_circ_0000567*	Down-regulated/ Tumor suppressor	qRT-PCR/2 − ΔΔCt	0.4714	*18S rRNA, GAPDH*	0.8653	Yes
Wang F 2018 [10]	Chinese	46	46	Pair-matched adjacent normal tissues	Tissue	*hsa_circ_0014717*	Down-regulated/ Tumor suppressor	qRT-PCR/2 − ΔΔCt	/	*GAPDH*	0.683	Yes
Wang X 2015 [11]	Chinese	62	62	Adjacent normal mucosa	Tissue	*hsa_circ_001988*	Down-regulated/ Tumor suppressor	qRT-PCR/ΔCt	6.04	*GAPDH*	0.788	Yes
Hsiao KY 2017 [12]		131	76	Paired noncancerous counterparts	Tissue	*circCCDC66*	Up-regulated/ Tumor promotor	RNA sequencing	/	*GAPDH*	0.88	Yes
Zhang P 2017 [13]	Chinese	170	170	Normal colorectal tissue samples	Tissue	*hsa_circRNA_104700*	Down-regulated/ Tumor suppressor	qRT-PCR/Ct	10.753	*GAPDH*	0.699	Yes
						*hsa_circRNA_103809*	Down-regulated/ Tumor suppressor	qRT-PCR/Ct	13.9	*GAPDH*	0.616	Yes
Li J 2018 [14]	Chinese	101	101	Paired noncancerous counterparts	Tissue	*hsa_circ_0000711*	Down-regulated/ Tumor suppressor	qRT-PCR/ΔCt	ΔCt: 3.37	*GAPDH*	0.81	Yes
Ji WX 2018 [16]	Chinese	64	64	Paired noncancerous counterparts	Tissue	*hsa_circ_0001649*	Up-regulated/ Tumor promotor	qRT-PCR	0.278	*GAPDH*	0.857	Yes
Li XN 2019 [19]	Chinese	60	60	Adjacent normal mucosa tissues	Plasma	*circVAPA*	Up-regulated/ Tumor promotor	qRT-PCR/2 − ΔΔCt	Median expression level of circVAPA	*U6*	0.724	Yes
Zhuo F 2017 [20]	Chinese	122	122	Paired noncancerous counterparts	Plasma	*circRNA0003906*	Down-regulated/ Tumor suppressor	qRT-PCR/2 − ΔΔCt	/	*GAPDH*	0.818	Yes

*AUC* area under the curve, *GAPDH* reduced glyceraldehyde-phosphate dehydrogenase, *qRT-PCR* quantitative reverse transcription-polymerase chain reaction

**Table 2:** Mian characteristics of the meta-analysis for prognosis and clinicopathologic association of circRNAs in CRC

Study	Locale	Case size		TNM Stage (I, II, III, IV)	Sample Type	CircRNA signature	Expression status/ Biological function	Survival indicator	Follow-up time	HR & 95% CI Extraction	Assessed clinicopathologic association
		High	Low								
Wang F 2018 [10]	China	23	23	I + II: 17, III + IV: 29	Tissue	*hsa_circ_0014717*	Down-regulated/ Tumor suppressor	OS	1 to 3 month intervals	Indirectly	Yes
Hsiao KY 2017 [12]	China	Total: 131		Unclear	Tissue	*circCCDC66*	Up-regulated/ Tumor promotor	OS	Unclear	Indirectly	Yes
Li J 2018 [14]	China	50	51	21, 32, 40, 8	Tissue	*hsa_circ_0000711*	Down-regulated/ Tumor suppressor	OS	Medain:39 month	Directly	Yes
Zeng K 2018 [15]	China	89	89	I + II: 121, III + IV: 57	Tissue	*circHIPK3*	Up-regulated/ Tumor promotor	OS	Unclear	Directly	No
Yuan Y 2018 [17]	China	15	17	Unclear	Tissue	*circ_0026344*	Down-regulated/ Tumor suppressor	OS	Unclear	Indirectly	No
Fang G 2018 [18]	China	24	20	Unclear	Tissue	*circRNA_100290*	Up-regulated/ Tumor promotor	OS	Unclear	Indirectly	No
Weng W 2017 [21]	China	76	77	19, 84, 47, 3	Tissue	*ciRS-7*	Up-regulated/ Tumor promotor	OS	Unclear	Directly	No
		89	76	26, 52, 49, 38	Tissue	*ciRS-7*	Up-regulated/ Tumor promotor	OS	Unclear	Directly	No

*OS* overall survival, *HR* hazard ratio

Study bias and quality assessed by QUADAS II and NOS checklists are shown in Tables 3 and 4. The rating scores of all eligible studies for diagnosis ranged from 4 to 6, and for prognosis ranged from 6 to 8, indicating high methodological quality in all included studies.

**Table 3:** Study quality of the diagnostic studies, as judged by the QUADAS II checklist

Study	Risk of bias				Concerns regarding applicability			Total stars
	Patient selection	Index test	Reference standard	Flow and timing	Patient selection	Index test	Reference standard	
Wang J 2018 [9]	Low	Unclear	Low	Low	Unclear	Low	Low	5
Wang F 2018 [10]	Low	Unclear	Low	Unclear	Unclear	Low	Low	4
Wang X 2015 [11]	Low	Unclear	Low	Low	Unclear	Low	Low	5
Hsiao KY 2017 [12]	Low	Unclear	Low	Unclear	Unclear	Low	Low	4
Zhang P 2017 [13]	Low	Unclear	Low	Unclear	Unclear	Low	Low	4
Li J 2018 [14]	Low	Unclear	Low	Unclear	Unclear	Low	Low	4
Ji WX 2018 [16]	Low	Unclear	Low	Unclear	Unclear	Low	Low	4
Li XN 2019 [19]	Low	Low	Low	Low	Unclear	Low	Low	6
Zhuo F 2017 [20]	Low	Low	Low	Low	Unclear	Low	Low	6

*QUADAS* Quality Assessment for Studies of Diagnostic Accuracy

**Table 4:** Study quality and bias in the retrospective cohort studies judged by the Newcastle-Ottawa Scale (NOS) checklist

Study	Total star	Cohort selection				Comparability	Outcome ascertainment		
		Representativeness of the Exposed Cohort	Selection of the Non-Exposed Cohort	Ascertainment of Exposure	Demonstration that Outcome of Interest Was Not Present at Start of Study	Comparability of Cases and Controls on the Basis of the Design or Analysis	Assessment of Outcome	Was Follow-Up Long Enough for Outcomes to Occur	Adequacy of Follow Up of Cohorts
Wang F 2018 [10]	8	1	1	1	1	1	1	1	1
Hsiao KY 2017 [12]	6	1	1	1	1	1	1	0	0
Li J 2018 [14]	8	1	1	1	1	1	1	1	1
Zeng K 2018 [15]	6	1	1	1	1	1	1	0	0
Yuan Y 2018 [17]	6	1	1	1	1	1	1	0	0
Fang G 2018 [18]	6	1	1	1	1	1	1	0	0
Weng W 2017 [21]	6	1	1	1	1	1	1	0	0

### Meta-analysis of clinical parameters

The association between circRNAs and clinicopathologic features in patients with CRC is shown in Table 5. Altered expression of circRNAs was markedly associated with poor clinicopathologic parameters (tumor diameter: pooled *P* = 0.0350; differentiation: pooled *P* = 0.0038; lymphatic metastasis: pooled *P* = 0.0119; distant metastasis: pooled *P* < 0.0001; TNM stage: pooled *P* = 0.0002; depth of invasion: pooled *P* = 0.0016). In contrast, no significant correlations were observed for age (pooled *P* = 0.3141), gender (pooled *P* = 0.5696), tumor location (pooled *P* = 0.8627), as well as levels of carcinoembryonic antigen (CEA) (pooled *P* = 0.2047), and carbohydrate antigen (CA) 19–9 (pooled *P* = 0.7954).

**Table 5:** Associations between circRNAs expression and clinicopathological features in CRC analyzed by Fisher’s test

Clinicopathological factors	Combined *P* value	*X*^2^ value	Enrolled Studies
Age	0.314135	20.33764	9
Gender	0.569616	16.32874	9
Cancer location	0.86275	3.937134	4
Diameter	0.035032	22.22902	6
Differentiation	0.003832	38.03542	9
Lymphatic metastasis	0.011944	22.69154	5
Distal metastasis	1.04E-05	37.23558	4
TNM stage	0.000229	33.44428	5
CEA level	0.204753	8.483865	3
CA19–9 level	0.795434	4.638385	4
Depth of invasion	0.001627	24.88387	3

### Diagnostic performance

**Fig. 2:**
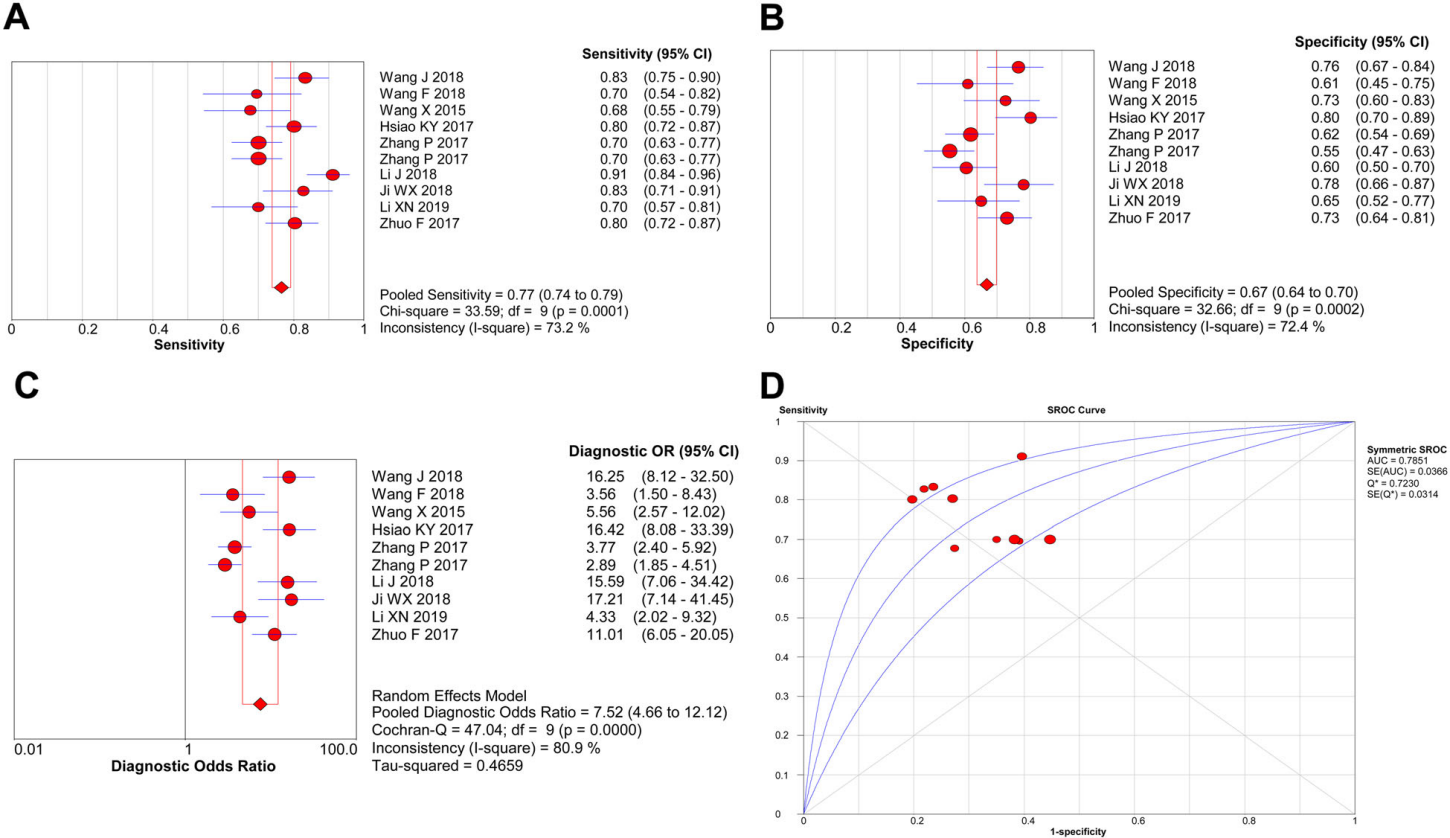
**a** Forest plots of the combined sensitivity, (**b**) specificity, (**c**) DOR, and (**d**) AUC for circRNAs expression in diagnosing CRC

The weighted diagnostic parameters of circRNAs in distinguishing CRC from non-tumor controls were as follows: sensitivity of 0.77 (95%CI: 0.70–0.82), specificity of 0.81 (95%CI 0.73–0.86), PLR of 4.00 (95%CI 2.80–5.60), NLR of 0.29 (95%CI 0.22–0.38), DOR of 14.0 (95%CI 8.0–24.0), and AUC of 0.86. Forest plots of the pooled sensitivity, specificity, DOR and summary receiver operating characteristic (ROC) curve of circRNAs in diagnosing CRC are shown in Fig. 2.

Stratified analysis showed that the performance of up-regulated circRNAs (function as tumor promoters) for CRC detection was significantly superior to that of down-regulated circRNAs (function as tumor suppressors) (AUC: 0.86 vs. 0.75; DOR: 10.63 vs. 6.55). When analyzed based on test matrix, these results showed that tissue-based circRNA testing achieved higher diagnostic efficacy than plasma-based analysis (AUC: 0.79 vs. 0.50; DOR: 7.68 vs. 7.13).

### Overall survival

**Fig. 3:**
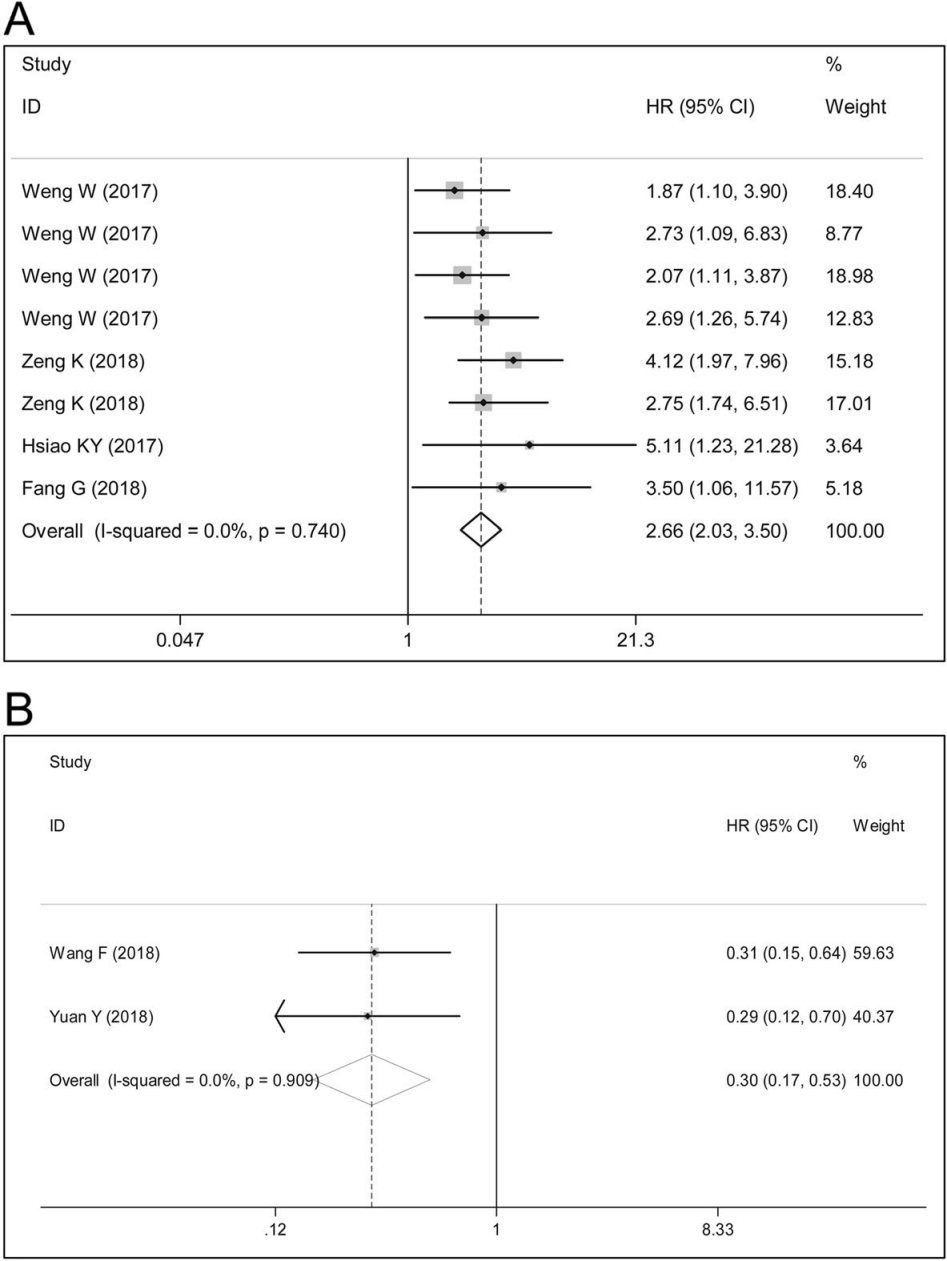
Forest plots of the combined HRs with 95%CIs respectively for the (**a**) up-regulated and (**b**) down-regulated circRNA profiles in predicting the overall survival (OS) of patients with CRC

**Fig. 4:**
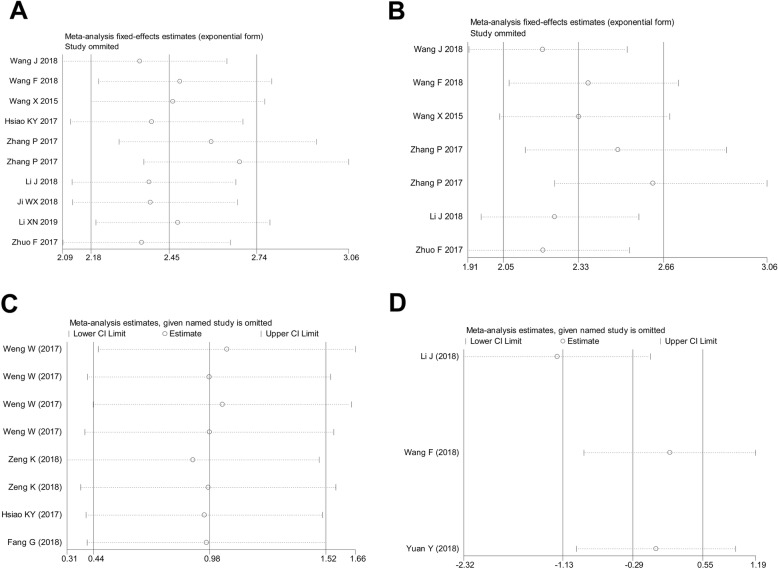
Sensitivity analysis of the outlier data for (**a**) the overall diagnostic studies, (**b**) the down-regulated circRNA profiles for diagnosis, as well as (**c**) the up-regulated, and (**d**) down-regulated circRNA expression signature in predicting the OS in CRC

Survival analysis showed that oncogenic circRNAs predict worse prognosis in terms of OS in patients with CRC (HR = 2.66, 95%CI: 2.03–3.50, *P* = 0.000; *Chi*^2^ = 4.34, *P* = 0.740, *I*^2^ = 0.0%) (Fig. 3). We identified one outlier study in the combined effect of decreased circRNAs by sensitivity analysis (Fig. 4), and the outlier data were eliminated. The weighted effect showed that decreased circRNAs expression (function as tumor suppressors) in patients with CRC was associated with favorable OS (weighted HR = 0.30, 95%CI: 0.17–0.53, *P* = 0.000; *X*^2^ = 1.34, *P* = 0.909, *I*^2^ = 0.0%) (Fig. 3).

### Sensitivity analysis and meta-regression

Results of the sensitivity analysis showed that the effect did not alter when omitting studies one by one in relation to the combined diagnostic effect and prognostic effect of oncogenic circRNAs (Fig. 4).

To identify the causes of heterogeneity, meta-regression of the pooled diagnostic effect in terms of the specified covariates such as sample size, text matrix, circRNA signature, expression status, reference gene, and quality score was conducted. The results showed that circRNA expression status (pooled DOR = 3.67, 95%CI: 1.10–12.28, *P* = 0.0386), and reference gene (pooled DOR = 0.29, 95%CI: 0.09–0.91, *P* = 0.0383) were likely to be the sources of heterogeneity (detailed data not shown).

### Publication bias

**Fig. 5:**
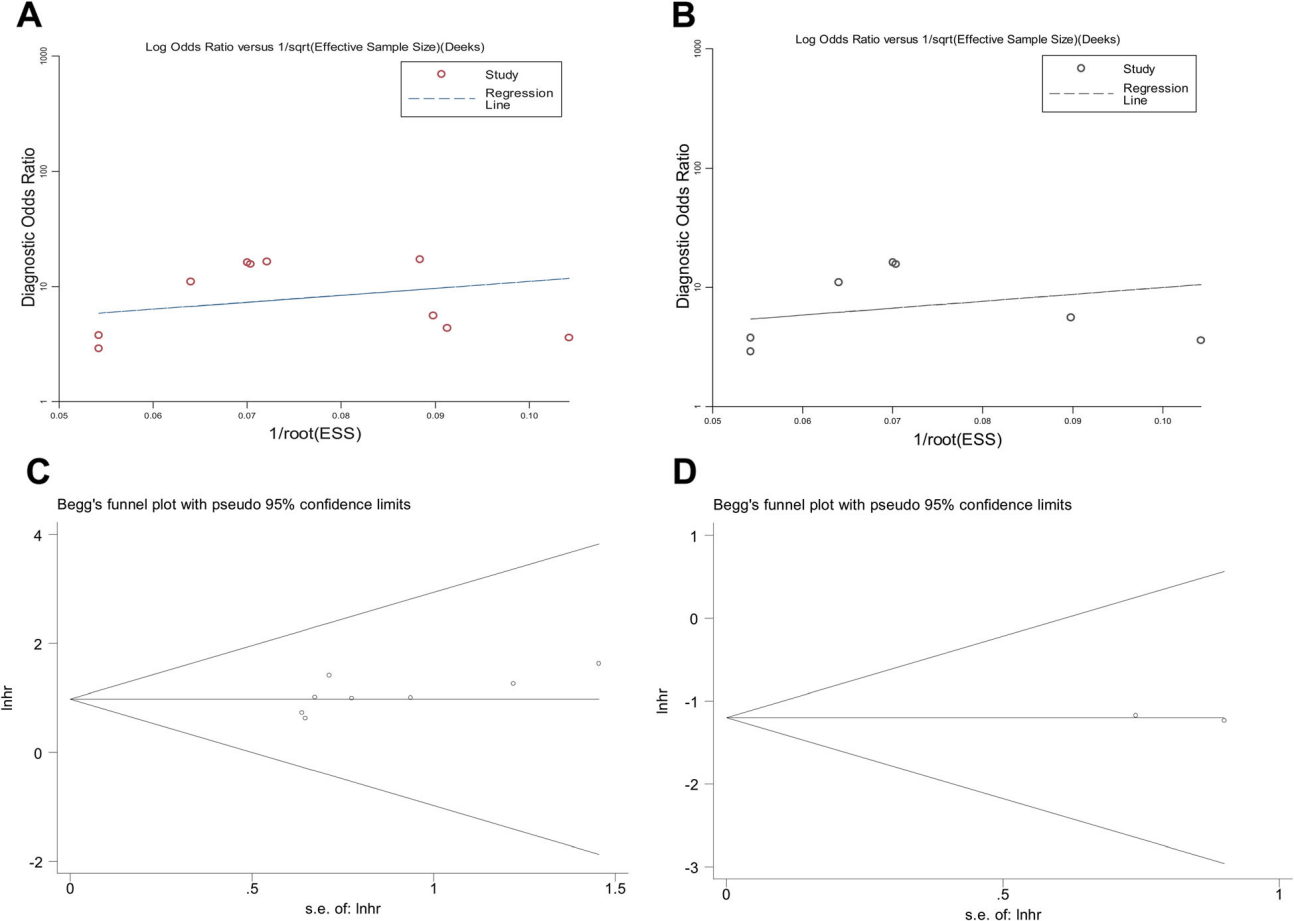
Publication bias assessed by the Deek’s funnel plot for (**a**) the overall diagnostic effect, and (**b**) the down-regulated circRNA profiles for diagnosis. Begg’s test for the (**c**) up-regulated, and (**d**) down-regulated circRNA expression profiling in predicting the OS in CRC

Deeks’ funnel plot asymmetry test showed that no evidence of publication bias (*P* = 0.37) existed for diagnostic analyses (Fig. 5a and b). Begg’s and Egger’s tests were also performed to assess publication bias among the eligible articles. There was no obvious publication bias in the prognostic effects according to Begg’s test (*P* = 0.129, or 0.266) (Fig. 5c and d), and Egger’s test (detailed data not shown). Therefore, we excluded the possibility of publication bias.

## Discussion

CRC is a major cause of cancer-related deaths worldwide [1–4]. The development of novel diagnostic and prognostic biomarkers to aid the clinical management of CRC is crucial. CircRNAs have been widely recommended as novel diagnostic and prognostic biomarkers in cancers, especially in CRC [9–21]. However, there are no relevant meta-analyses focused on circRNAs expression in CRC. This study systematically analyzed the clinical, diagnostic, and prognostic significance of abnormally expressed circRNAs in CRC.

Studies have suggested a marked relationship between circRNAs expression and CRC [9–11, 13, 14, 16, 19, 20]. In the present study, abnormally expressed circRNAs were found to be associated with tumor diameter, differentiation, lymphatic metastasis, distant metastasis, TNM stage, and depth of invasion, suggesting that dysregulated circRNAs are implicated in the progression of CRC. Significant correlations were not found for age, gender, tumor location, CEA and CA19–9 levels.

The ROC curve is a comprehensive index, which reflects the sensitivity and specificity of continuous variables [27, 28]. Our summary outcomes revealed a moderate diagnostic efficacy for circRNAs expression in CRC, and diagnostic sensitivity was estimated to be 0.77, and specificity was 0.67. The pooled AUC of circRNAs indicated that 78% of randomly chosen CRC patients would have lower or higher levels of circRNA(s) than normal controls. The pooled DOR is also an important indicator that facilitates formal meta-analysis of studies on diagnostic test performance [29, 30]. In the present study, a pooled DOR of 7.52 (higher than 1.0) was obtained, suggesting that dysregulation of circRNA expression is a powerful predictive biomarker for CRC diagnosis. As circRNAs with different expression status may exert different functions in CRC, we conducted subgroup analyses. Stratified analysis based on circRNA expression status showed that circRNAs, which function as tumor promoters, yielded higher efficacy than tumor suppressors, and tissue-based circRNA analysis showed higher diagnostic efficacy than plasma-based analysis. However, the sample size was reduced in the subgroup analyses; thus, the accuracy was compromised. Moreover, when taking conserved sequences and stable structures into consideration, circRNAs may serve as novel noninvasive biomarkers in CRC detection.

 Studies have documented that circRNAs with dysregulated expression are emerging as independent risk factors for OS in cancer [31, 32]. Consistent with these data, our pooled effect sizes demonstrated that oncogenic circRNAs overexpression was strongly correlated with decreased OS time in patients with CRC (HR = 2.66, *P* = 0.000). With regard to the prognostic significance of down-regulated circRNAs (may function as tumor suppressors), we identified one outlier study in the combined effects of decreased circRNAs by sensitivity analysis, and the weighted effect showed that decreased circRNAs expression was associated with improved OS in patients with CRC (HR = 0.30, *P* = 0.000).

To identify the cause of study heterogeneity, we first performed sensitivity analysis. The results showed that studies were relatively homogeneous in the overall combined diagnostic effect and prognostic effect of oncogenic circRNAs. However, we identified an outlier study in the pooled prognostic significance of down-regulated circRNAs; thus, the effect size was weighted. On the one hand, the meta-regression test further showed that circRNA expression status and the reference gene were likely to be the sources of heterogeneity. We included 13 types of circRNAs with a different expression status in CRC, and the quantitative analysis was based on different reference genes (*GAPDH, 18S rRNA*, or *U6*); therefore, the heterogeneity was generated in the pooled effects. On the other hand, neither the Deeks’ funnel plot asymmetry test nor the Egger test and Begg’s funnel plot revealed obvious publication bias for the diagnostic and prognostic meta-analyses, suggesting that all pooled effect sizes were reliable.

Several limitations should be acknowledged in our study. Firstly, although we combined individual studies and increased the number of cases, heterogeneity was observed in some combined effects. Secondly, the small sample size in sub-group analyses as well as the indirectly extracted HR values may increase the insufficiency of statistical power. Finally, population bias may exist in our analyses as most of the studies were conducted in China.

## Conclusions

In summary, the results of the meta-analysis revealed that circRNAs are promising diagnostic and prognostic biomarkers in patients with CRC, and may therefore serve as therapeutic target(s). Further prospective studies on more types of circRNAs are warranted in the future.

## Data Availability

The data that support the findings of this study are available on request from the corresponding author.

## Abbreviations

AUC: Area under the curve
CRC: Colorectal cancer
DOR: Diagnostic odds ratio
HR: Hazard ratio
NLR: Negative likelihood ratio
NOS: Newcastle-Ottawa Scale
OS: Overall survival
PLR: Positive likelihood ratio
QUADAS: Quality Assessment for Studies of Diagnostic Accuracy
